#  Brain Tumor-Related Epilepsy

**DOI:** 10.2174/157015912800604470

**Published:** 2012-06

**Authors:** Marta Maschio

**Affiliations:** Center for Tumor-Related Epilepsy, Neurology Unit, Department of Neuroscience and Cervical-Facial Pathology, National Institute for Cancer “Regina Elena” Via Elio Chianesi, 53 00144 Roma, Italy

**Keywords:** Antiepileptics, brain tumor-related epilepsy, chemotherapy, epilepsy, pharmacological interactions, quality of life, side effects.

## Abstract

In patients with brain tumor (BT), seizures are the onset symptom in 20-40% of patients, while a further 20-45% of patients will present them during the course of the disease. These patients present a complex therapeutic profile and require a unique and multidisciplinary approach. The choice of antiepileptic drugs is challenging for this particular patient population because brain tumor-related epilepsy (BTRE) is often drug-resistant, has a strong impact on the quality of life and weighs heavily on public health expenditures.

In BT patients, the presence of epilepsy is considered the most important risk factor for long-term disability. For this reason, the problem of the proper administration of medications and their potential side effects is of great importance, because good seizure control can significantly improve the patient’s psychological and relational sphere.

In these patients, new generation drugs such as gabapentin, lacosamide, levetiracetam, oxcarbazepine, pregabalin, topiramate, zonisamide are preferred because they have fewer drug interactions and cause fewer side effects. Among the recently marketed drugs, lacosamide has demonstrated promising results and should be considered a possible treatment option.

Therefore, it is necessary to develop a customized treatment plan for each individual patient with BTRE. This requires a vision of patient management concerned not only with medical therapies (pharmacological, surgical, radiological, etc.) but also with emotional and psychological support for the individual as well as his or her family throughout all stages of the illness.

## INTRODUCTION

Patients with brain tumor related epilepsy (BTRE) present a complex therapeutic profile and require a unique and multidisciplinary approach. There are many factors to take into consideration. First, there is the management of pharmacological therapies: the concurrent use of antiepileptic drugs (AEDs), chemotherapy (CT), and support therapies can present problems with drug interactions and collateral effects [1].

Secondly, there is the concern for maintaining a good quality of life (QoL) for these patients. 

In addition, we must recognize the fact that epilepsy still brings with it stigma and can cause the individual who is diagnosed with the disease to feel socially outcast and severely invalided.

Considering all of these factors, it is understandable that freedom from seizures or at least a reasonable control of them, is of utmost importance for the patient, if he/she is to resume his/her professional life, function successfully in a social context, and conduct a satisfying family life. For thisreason, it is fundamental that healthcare professionals see the patient as a unique individual with his/her particular needs. This requires a vision of patient management concerned not only with medical therapies (pharmacological, surgical, radiological, etc.) but also with emotional and psychological support for the individual as well as his or her family throughout all stages of the illness.

### Brain Tumors

#### Incidence

Improvement of life expectancy with BT is associated with the histological classification as well as with a younger age. Many medical institutions use the classification system of the World Health Organization (WHO) to identify BTs. The WHO system classifies tumors according to their original cell make-up and according to the way they “behave”, starting with the least aggressive (i.e. benign) and moving to the most aggressive (i.e. malignant) [2,3]. 

The overall incidence of BTs is 18.71% cases per 100,000 inabitants/yearly. Benign tumors: 11.52% cases per 100,000 inabitants/yearly. Malignant tumors: 7.19% cases per 100,000 inabitants/yearly; these tumors represent 1,1 – 2% of all adult tumors and are considered rare [4,5]. Brain metastases are one of the most common neurologic complications of cancer. The incidence is 9%-17% based on various studies, although the exact incidence is thought to be higher [6]. The incidence is increasing due to improved imaging techniques that aid early diagnosis and effective systemic treatment regimens, which in turn prolong life, thus allowing cancer to disseminate to the brain. Brain metastases are most frequent in lung cancer, breast cancer, and melanoma and account for 67-80% of all cancers [6].

The incidence of primary tumors of the central nervous system in Europe is 5 cases per 100,000 inabitants/yearly (3.7 per 100,000 persons/year for men and 2.6 per 100,000 persons/year for women) without significant differences among single European nations, resulting in 2% of all cancer-related deaths. Over the last 3 decades, there has been a progressive increase in the incidence of these tumors: this increase is not attrubuted to the mere development of more accurate diagnostic methods (neuroradiological exams) that facilitate earlier diagnosis, but is problably due to other causes not yet clearly understood. The most significant increase has been seen among individuals over the age of 65, where the incidence has more than doubled [7-10].

Glioma tumors represent 67.6% of all primitive BTs, and derived from three types of glial cells: astrocytes (astrocytomas), oligodendrocytes (oligodendrogliomas) and ependymal cells (ependymomas). They have the following characteristics: the impossibility of a complete surgical resection, incurability, genetic instability, progressive worsening over time, tendency towards local reoccurance after the initial therapy. They are responsible for more than 26,000 deaths per year in the United States and Europe [11]. All gliomas have the potential to become malignant neoplasms through a process af malignant degeneration. The glioblastoma multiforme (GBM) is the most aggressive form of BT and the most frequent, with an incidence of 7-8 cases for every 100,000 inhabitans. Of these types of tumor, one third is diagnosed in patients age 60 and older [8,12]. GBM represent 45–50% of all gliomas, age of onset 45 – 65 years of age, and ratio male-female 1.5:1. The median survival is 12-18 months for primary GBM. Anaplastic astrocytoma are more frequent in younger patients and represent 10-35% of all gliomas. The age of onset is 35-55 years. Ratio male-female 1.2:1 and a median survival of 24-36 months to 5 years [13,14]. Low-grade gliomas are more frequent among individuals between 20 and 40 years old, while high-grade gliomas generally appear later, between 40 and 70 years of age. Age, perfomace status (i.e. degree of personal autonomy), and histological grade are the most significant prognostic factors with regard to gliomas. Gliomas stage II are slow growing tumors, with variable tendency towards increasing malignancy and, in most cases, an unfavorable prognosis (median life expectancy around 5 years for astrocitomas, 9–10 years for oligodendrogliomas, and intermediate for mixed tumors). Epilepsy is of particular significance in the management of young patients with low-grade tumors, who have a discrete prognosis, because of their active social and professional lives, where the lack of seizure control and possible side effects of antiepileptic drugs can seriously compromise their QoL [15].

### Quality of Life

The histological type of tumor, type of anti-neoplastic treatment (CT, radiation), type of support therapy (corticosteroids, antiacids, neuroleptics, etc.), antiepileptic drug, and other possible medical complications can affect the patient’s neurocognitive functioning, psychological well-being, and the ability to perform daily tasks. However, much can be done to improve the patient’s QoL and his/her assistance. An understanding of how the effect of the tumor itself, various therapies and host of other factors, can influence functions of the brain as well as the QoL of the patient, guides the development of all inclusive therapeutic strategies that take into account all of these concerns. The impact that a BT can have on the individual’s daily performances is reflected well by the World Health Organization’s three level system. 

#### Impairment,

refers to the deficit in cerebral functioning caused by the illness and is determined using neurological and neuropsychological evaluations. 

#### Disability

refers to the impact of the patient’s deficit with regard to performing certain activities and is evaluated using the Performance Status and functional status.

#### Handicap

is the impact of the disability on the patient’s subjective well-being and on social functioning and is generally determined using clinical evaluations and QOL questionnaires [2,3]. 

### Brain Tumor-related Epilepsy

#### Incidence

The most common symptom in patients with BTs is epilepsy. In patients with BT, seizures are the onset symptom in 20-40% of patients, while a further 20-45% of patients will present them during the course of the disease. Overall, the incidence of epilepsy in BTs, regardless of histological type and anatomical site of the lesion varies from 35 to 70% [7,12,16-20]. Epilepsy due to BTs constitute 6-10% of all cases of epilepsy as a whole and 12% of acquired epilepsy [21,22]. 

Seizures appear in 20-40% of patients with brain metastases, especially when there are multiple metastases. Of patients who do not have seizures as a presenting symptom, 10% will develop them during the course of the disease. Seizures appear in 67% of patients with brain metastases from melanoma, in 48% with lung cancer, in 33% with breast cancer and in 55% with unknown cancer [1,23-30].

Brain tumor-related epilepsy is charaterized by its pharmacological resistance. Resistance to AEDs is defined as continuous episodes (seizures) despite treatment with at least three tolerated AEDs which were appropriately used at maximum dosage [14]. Pharmacoresistant Epilepsy can be classified as: *primary* (related to intrinsic component of the illness) or *secondary* (e.g. undesired consequences of the illness itself), or *specific* (e.g. due to a response to a specific drug) or *not specific* (e.g. due to a response to a variety of drugs).

Based on these classifications, BTRE can be considered a pharmacoresistent epilepsy with mixed characteristics: primary (presumed to be related to the tumor itself); secondary (limited efficacy of pharmacological therapies due to drug interactions) and often not due to only one specific drug). Actually, the related pathophysiological mechanisms that give rise to epileptic seizures in BT patients are unclear [31,32], while the correlation of seizures with other factors such as histology, site of tumor, and age of onset, have been well documented in the literature. 

The incidence of epilepsy onset is inversely correlated to malignancy, with the highest incidence (from 65-95%) occurring in low-grade tumors (astrocytoma, oligodendroglioma and mixed astrocytome I and II WHO, and meningiomas), and the lowest incidence (from 15-25%) occuring in malignant gliomas [8,14]. 

Epilepsy onset at a young age is more frequent for slow growing tumors and is associated with a higher probability of epilepsy. A very important factor which determines whether or not there will be seizures is the location of the tumor, with a higher seizure frequency being associated with supratentorial tumors (with respect to subtentorial tumors), located in the cortex and superficially [11].

The onset of seizures seems to indicate a more favorable prognosis [16]: this could be due to early diagnosis resulting from following up on the manifestations of seizures, to a better position of the tumor for surgical intervention (a more superficial location), or to the presence of a more favorable hystology (slow-growing tumors). 

Epilepsy can be a symptom not only of the site of the tumor, but also of the tumor’s biological development. In fact, though the role genetics play in BTRE is unknown, several hypotheses could be made suggesting common pathways between the two (i.e. the tumor and epilepsy). Particularly, in reference to the relationship between the onset of seizures in BT and the role of certain neurotransmitters, such as GABA and glutamate. Some experimental studies have pointed to a dysfunction of pre and post-synaptic GABA-B receptors of the temporal lobe in epilepsy and have also indicated a possibile role of GABA in the genesis of gliomas [33].

In addition, the expression of benzodiazepine receptor in the brain seem to be correlated to the histological stage of malignancy of gliomas; GABA seems to have a direct immune-modulatory role in the brain [33,34]. Regarding the role of glutamate, its role in the genesis of gliomas as well as in BTRE has been widely accepted. In fact, glioma cells release glutamate which causes the death of neurons in surrounding tissue. This seems to be one of the mechanisms at the root of invasive growth of the tumor [33,35]. 

This data is of great importance due to the fact that BTRE is often drug-resistant, has a strong impact on QoL of patients, and weighs heavily on public health expenditures. Therefore, in order to choose the correct treatment in this particular patient population, it is critical to take into account the possible mechanisms of drug resistance and tumoral epileptogenesis.

#### Farmacoresistance and Epileptogenesis

The pathophysiologic mechanisms of seizures in patients with BTs remain unclear and appear to be multifactorial, related to mechanisms that bypass AEDs [31,32]. Poor seizure control may result from the fact that the antiepileptic action of many AEDs is due to a modification of membrane excitability mediated by ion channels, but mechanisms of epileptogenesis in BTRE include also changes in pH, amino acids, proteins, etc. Seizures in BTRE may also be due to tumor progression (in this case, the first AED is often not sufficient) or to a low concentration of AEDs in serum or site of action that can result from drug interactions with cancer therapies [12,20,36-40]. In patients with drug-resistant epilepsy, a reduced intraparenchymal accumulation of AEDs has been demonstrated. This phenomenon can be due to over-expression of genes and proteins that mediate nonspecific resistance to treatment. These proteins have also been found in neurons and glia of the epileptogenic zone. In patients with BTRE, the growth of these intracellular proteins may be caused by the tumor. Transport proteins were discovered by Victor Ling, in cancer cells resistant to CT and were named multidrug resistant proteins (MDR) or P-glycoprotein (P-gp). To date, there are several MDR P-gp, MRP1, MRP2, among others. The primary function of these proteins is to pump lipophilic xenobiotics out of cells to prevent the accumulation of potentially toxic substances. By doing so, the effectiveness of these drugs diminishes by restricting access to intra-cerebral target tissues [41-43]. Although there are many MRPs in the endothelial cells of the BBB, P-gp is the most important in pharmaco-resistant epilepsy as it is capable of carrying a large number of AEDs, including: carbamazepine (CBZ), felbamate, gabapentin (GBP), lamotrigine (LTG), phenobarbital (PB), phenytoin (PHT) and topiramate (TPM). It is likely that Pgp reduces the access of certain drugs such as PHT and levetiracetam (LEV) in human brains because as experimental data has demonstrated, the resistance to these drugs is associated with over-expression of Pgp and MRP2 to the blood brain barrier [43]. Also, the intra-cerebral concentration of oxcarbazepine (OXC) is inversely proportional to the expression of MDR1 mRNA in patients with refractory epilepsy.

#### Seizure Classification

The type of epileptic seizures, for the most part, are partial (simple or complex). Partial seizures with secondary generalization are also frequent, but they are difficult to recognize clinically [11,17]. Occasionally, repeated complex partial seizures (often originating in the temporal lobe) can cause a non-convulsive epileptic state with variable duration that can last up to several hours. The clinical manifestations of these types of seizures can take the form of a confusional state, automatisms or behavioral modifications, which from a clinical standpoint, can be confused with psychiatric disorders or other causes. Primary generalized seizures rarely occur in these patients [1]. In 73% of patients with BTs, epilepsy can be the presenting symptom or can occur during the course of the oncological disease due to a number of factors: 1) directly relating to the reoccurance of brain cancer or to disease progression; 2) directly related to the therapies (CT, support therapies and radionecrosis) or for other causes such as vascular, intective, metobolic, and limbic encephalopathy [18].

Brain tumor-related epilepsy does not represent a simple sum of many challenges -- therapeutic, support and psychosocial, that result from having two serious pathologies simultaneously: on one hand the brain-tumor and on the other hand, epilepsy. A diagnosis of BT and just the idea of cancer alone, in most patients, is enough to cause grave difficulties: behavioral, emotional, and intellectual. These problems can lead to a compromised daily life and a limited ability to live indipendently. Depending on the site of the lesion on the brain, there can be serious neurocognitive disturbances, for example: fatigue, depression, and cognitive deficits; with the latter often comprimising functional independence of the patient more than a physical disability, and therefore considered a factor which can significantly influence QoL and therapeutic choices [44]. In addition to the site of the lesion, cognitive functioning can also be affected by systemic therapies (CT and radiotherapy - RT) [45].

To date there have been several reports concerning the impact of systemic treatment on seizure reduction [45]. The percentage of seizure-free patients after tumor surgery ranges from 65 to 82%. The most significant factors associated with seizure freedom are completeness of tumor resection and short preoperative duration of BTRE [45]. The effect of CT on seizure frequency is obtained in 50-65% of patients with 20-40% of seizure freedom [45], but it is important to note that the main objective of the these studies was not the evaluation of BTRE, but clinical oncological response. Few studies have analyzed the impact of RT on BTRE showing that RT has an efficacy on seizure control ranging between 40-100% depending on different RT technique (gamma-knife, conventional RT, radiosurgery) [45]. 

#### Brain tumor-related Epilepsy and Quality of Life

The diagnosis of epilepsy in a patient with no oncological disease already implicates an important change in one’s concept of QoL, that involves three main factors:

1. Possibile side effects from drugs.
2. The negative psychological impact caused by losing control of one’s body and the surrounding environment during seizures.
3. The rejection and marginalization that still occurs today due to a societal view of individuals with epilepsy as “strange”.

These three factors become even heavier to bear in patients that must confront both pathologies: epilepsy and BT. These patients are subjected to systemic treatments for the neoplastic disease as well as antiepileptic therapies, and therefore are at even greater risk for side effects and drug interactions. The loss of control of one’s body during a seizure and the frustration that accompanies such an experience, represent for the patient a total lack of autonomy. The unpredictability of adverse events leads to an enormous sense of insecurity. In addition, seizures are a constant reminder to the patient of his/her illness and of being considered “different”. Marginalization and rejection are especially felt by individuals who have a visible physical disability like hemiparesis or problems with speech (which may be due to the site of the tumor), and also by those whose physical aspect has been altered due to systemic therapies (hair loss from radiation, ritention of liquids, or noticeable weight gain due to the assumption of steroids). All of these factors together with the label “epileptic” can cause the patient to feel extremely frustrated when attempting any type of social and/or interpersonal relationship. Taking into consideration all of these factors, it is understandable why total seizure freedom or a least a good control of seizures is so essential to the patient’s ability to resume work and normal family and social relationships. 

#### Impact of New AEDs 

The presence of epilepsy is considered the most important risk factor for long-term disability [44,46] in BT patients. For this reason, the problem of the proper administration of medications and their potential side effects is of great importance. Good seizure control can significantly improve the patient’s psychological and relational sphere (i.e. social, personal, and professional). Many studies (meta-analyses) pertaining to epileptic patients have been done, but it is difficult to transfer the results to clinical practice [15,47-50]. 

##### 1. Effectiveness 

Regarding the clinical efficacy of AEDs in BTRE patients, studies with the old AEDs such as CBZ, PB, and PHT are few and offer conflicting data. Data on the efficacy of new AEDs on complete seizure control, in monotherapy or as add-on, are documented in recent studies. In particular: OXC monotherapy: 62.9% patients seizure-free; TPM monotherapy: 55.6% patients seizure-free, GBP, pregabalin (PGB), tiagabine (TGB), and zonisamide (ZNS) in add-on: responder rate from 27.4 to 100%; LEV both in monotherapy and as add-on: patients ​​seizure-free from 47.4% to 88% [51-56]. Only one study on lacosamide (LCM) as add-on with 42.9% patients seizure free [57].

##### 2. Side Effects

The adverse effects of a drug can be divided into two types: i) idiosyncratic toxicity which is dose-independent, unpredictable, and usually manifests itself in the early stages of assumption of the drug; ii) acute toxic effects, which are dose-dependent, very frequent, and can occur throughout the course of treatment with the drug (these effects are often due to pharmacokinetic modifications induced by concomitant therapies), and iii) chronic toxic effects, which instead arise after months or years of treatment, are linked to the total quantity of the drug consumed, are specific for each drug, and can even appear at therapeutic dosages. 

Adverse effects of AEDs are more frequent in patients with tumor-related epilepsy than in the rest of the epileptic population [16,18]; a recent meta-analysis [16] in fact showed the appearance of adverse effects severe enough to warrant suspension or modification of the AED therapy in 24% of patients affected by tumor-related epilepsy, as opposed to 0.5-12% of patients without tumor. In particular, many AEDs, in addition to the idiosyncratic, haematological, and systemic toxicity effects, also have effects on the central nervous system (CNS), which can strongly impact the patient’s QoL, make it difficult to correctly assess the response to CT, and even mimic a progression of the tumour [7]. A recent study showed that cancer patients who use CBZ, PB, valproic acid (VPA) and PHT showed worse cognitive performances, with the exception of verbal memory, than those who do not use them [15]. Every AED is associated with certain adverse effects, in both oncological and non-oncological patients. However, some of these can assume particular significance in the cancer patient:

1. PB seems to be associated with the worst cognitive profile (sedation, behavioural problems, cognitive deficits, depressed mood) [15], and its use is thus not recommended in patients with brain tumour and cognitive deficits [58]. It can also cause megaloblastic anaemia and scapular-humeral periarthritis: the latter often causes pain and functional impotence, which can aggravate the tumor-related disability.
2. CBZ can cause dizziness, diplopia and sedation, whereas its most feared idiosyncratic effect, albeit rare, is haematological toxicity; it can also cause, just at the start of treatment, a mild and non-progressive leucopenia, which does not necessitate suspension of the drug.
3. PHT rarely gives rise to idiosyncratic reactions; it can cause agranulocytosis (which does require suspension of the drug) and acute encephalopathy with psychological and neurological problems that, in the absence of the classic signs of toxicity, can seem to suggest a progression of the tumour. Combined use of PHT or CBZ during RT seems to be associated with a higher risk of developing severe cutaneous reactions (even Stevens-Johnson syndrome), a risk that must be taken into account by physicians prescribing these drugs to neuro-oncological patients about to undergo RT [59].
4. VPA can, in some cases, cause acute encephalopathy whose symptoms may suggest a progression of the tumour. It can induce coagulation deficits and thrombocytopenia (and thus worsen the thrombocytopenia caused by the chemotherapeutic agents). Increased haematological toxicity has also been reported in combined therapy with VPA and nitrosoureas [60].
5. OXC does not significantly alter cognitive function; in some cases it even seems to improve psychomotor functions, in particular, attention and manual writing speed [61-63]. The most frequent CNS-related adverse effects, usually only moderate, are somnolence, headache and dizziness. Although OXC therapy has been associated with hyponatraemia, this is usually asymptomatic and does not necessitate suspension of the drug [63].
6. TPM can cause problems with language and memory. In particular, one double-blind study of healthy subjects showed a global deterioration of cognitive functions, especially language and memory, but not of motor performances [64]. Conversely, a study in patients with epilepsy showed that TPM administered as an add-on therapy to CBZ was well tolerated and did not produce appreciable cognitive adverse effects [65,66].
7. The most frequent adverse effects of LTG are CNS-related (headache, diplopia, nausea, ataxia, dizziness), but it can also cause rashes, eosinophilia and Stevens-Johnson syndrome [67].
8. Among the new AEDs, VGB, GBP and LEV are not metabolized by oxidation or conjugation: therefore they show little or no interaction with other drugs. Vigabatrin, however, can cause sedation, depressed mood and psychoses [68], as well as severe visual disorders. Gabapentin has few adverse effects [69], but its efficacy in controlling seizures has not, to date, been proven. Levetiracetam appears to be well tolerated, showing good efficacy and few adverse effects, but it can cause aggressive behaviours and agitation [70].

In short, all the GABAergic drugs (PB, benzodiazepine, VGB, TGB and TPM) have sedative effects and can induce depression; VPA and LTG, on the other hand, have antidepressant properties [1]. In addition, PB, PHT and CBZ are osteopenic, and thus associated with an increased risk of fractures, particularly of the hip and heel, whereas VPA is associated with reduced bone density [71].

In cancer patients, the evaluation of side effects (SE) of an AED is crucial due to the fact that SE can affect the patient’s perception of QoL more than seizure frequency [15]. Patients’ priorities often have less to do with seizure freedom, than with the desire to have the least amount of SE induced by drugs which they perceive of as being extremely limiting on their daily lives. With the older AEDs, there is a high incidence of serious SE (23.8%) and a mean of incidence of SE (20-40%), higher than in the non-oncological epileptic population [16]. Only recently, studies have been published that have evaluated the percentage of side effects that appeared in BTRE patients. These data with respect to the new AEDs indicated a percentage of side effects of 7.1% with LCM (blurred vision, dizziness), 11.4% with OXC (rash), 14.9% with TPM, (cognitive disturbances, weight loss), 22 to 37% with LEV, (somnolence, restlessness), 33.3% with ZNS (erectile dysfunction, drowsiness) and 55.5% with PGB (fatigue, oedema) [51-57,72-78]. Of all of the possible SE that can appear in BTRE patients undergoing AED therapy with the newer drugs, rash is potentially very serious. However, to date, there is only one case report in the literature [78] documenting the appearance of rash in 4 patients with BTRE in mono-therapy with the new AED, OXC, during RT. This indicates that the risk of serious skin reactions in patients treated with AEDs during RT should not be underestimated, even with the use of new AEDs [78].

In patients with BTRE, SE of new AEDs can influence two particolar aspects of their QoL: cognitive functions and sexual sphere.

##### 2a. Impact on Cognitive Functions

There have been no studies dedicated specifically to studying the impact of the older AEDs on cognitive function in patients with BTs. However, there have been studies on cognitive function in oncological patients in general [15,79-81]; while not examining the impact of AEDs in this specific area, these studies demonstrated that the older AEDs such as PHT CBZ VPA and PB have the highest incidence of adverse effects on cognitive function. Few studies on the cognitive and psycho-social effects of the newer AEDs in BTRE patients are present in the literature, but one recent study demonstrated the positive effect of a new AED, LEV, on social function and personal interaction [54]. 

##### 2b. Sexual Dysfunctions

Last, but not least, we must not forget that BTs often affect young people, for whom a satisfactory sexual relationship can be a fundamental aspect of emotional well-being, and a good QoL. For this reason the choice of the AED should take into account the possible effects on sexuality [82,83]. In patients with epilepsy, percentages of sexual dysfunction is 11-22% during treatment with PB, CBZ, PHT, primidone (PRM). There have been only two studies published in which two cases of reversible sexual dysfunction are described using a newer AED, TPM [84,85].

To date there are no randomized trials or comparison on the effect of AEDs (both old and new) on sexual satisfaction in patients with BTs. The only case report in the literature [86] concerns an erectile dysfunction related to the assumption of add-on ZNS in a patient with oligoastrocytoma. This symptom completely disappeared upon withdrawal from this drug. 

##### 3. Pharmacological Interactions

A pharmacological interaction occurs when one drug modifies the activity of another, increasing or reducing its effects [37,87]. Pharmacokinetic interactions occur when one drug interferes with the distribution in the organism of another drug, altering its concentration at the site of action. This results in changes in the plasma levels of both drugs and of their metabolites. These interactions can occur at any stage during the drug’s passage through the organism [88]. In the drug absorption phase, some drugs can alter the absorption of others. For example, antacids reduce absorption of PHT, PB, CBZ and GBP, by reducing gastric acidity and forming complexes that are insoluble with them. During the elimination phase, a drug is eliminated from the organism through renal excretion (as in the case of VGB, GBP, LEV and felbamate), or hepatic excretion. Elimination by the hepatic excretion route, which is associated with the most clinically significant pharmacological interactions, may occur by means of glucuronidation (as in the case of LTG) or by means of biotransformation of the drug mediated by CYP isoenzymes belonging to the cytochrome P450 system. These isoenzymes catalyze the oxidation reactions both of drugs and of endogenously produced substances, and their function can be modified by the concomitant administration of other substances [38,89]. This means that P450-enzyme-inducing drugs provoke faster elimination of a second concomitantly administered drug and thus a reduction of its efficacy, or at least of its concentration at the site of action. CYP-enzyme-inhibiting drugs decrease the metabolism of the second drug, increasing the risk of toxicity and of the appearance of adverse effects. Of the AEDs, PB, PRM, CBZ and PHT act as enzyme inducers, and thus enhance the metabolism of corticosteroids (drugs routinely administered to patients with BTs) and of many chemotherapeutic agents, including nitro-soureas, paclitaxel, cyclophosphamide, etoposide, topotecan, irinotecan, thiotepa, adriamycin and methotrexate. This means that the administered doses of these drugs could be inadequate, and therefore less effective [16,18,37] (Table **1**). On the other hand, costicosteroids and many chemo-therapeutic agents, too, are inducers of the CYP enzymes and can therefore alter the efficacy or increase the toxicity of many AEDs administered concomitantly. There are, to date, no data in the literature suggesting that the new AEDs interfere with anti-tumor drugs [37].

The dynamic involved in possible drug interactions appears to be related to the stage of the disease and to the pharmacological therapies associated with the illness. In fact, CT with temozolomide given to patients with primary BT, is not significantly metabolized by the CYP450 hepatic system, thus limiting the possibility of drug interactions. On the other hand, the so-called “rescue therapies” (erlotinim, imatinib, cediranib) have a purely hepatic metabolism and are rapidly metabolized when used in combination with enzyme-inducing drugs. They often require a triple dose in order to maintain a constant plasma level of CT, which leads to higher costs and potential health hazards in the event of a non compliant patient who discontinues AED therapy [36,89,90]. 

In patients with epilepsy related to brain metastases from solid tumors, CT such as irinotecan, taxanes, vinca alkaloids and teniposide are used, all of which have a hepatic metabolism and in combination with enzyme-inducing AEDs are rapidly metabolized, thus resulting in decreased efficacy, which clinically translates into reduced survival of patients [40,60,89,90-92]. 

The possible antitumor effect of VPA has been hypothesized due to an *in vitro* action on hystone deacetylase which has shown that it could induce growth arrest through the promotion of apoptosis, the reduction of differentiation and suppressed colony-forming efficiency and tumorigenicity [93,94]. However, this has not been sufficiently confirmed in clinical practice [93]. One retrospective study (which did not have as primary objective the evaluation of the activity of the AEDs), highlighted an advantage with regard to survival rate in patients treated with VPA and temozolomide, suggesting a possible synergy between VPA and the CT [95]. In fact, in contrast to this theory, the use of VPA in patients receiving cisplatin and nitrosoureas has demonstrated incidence of thrombocytopenia and neutropenia or platelet dysfunction [7,60].

The possible long-term disability that epilepsy can provoke in BTRE patients necessitates that the choice of AED takes into consideration: the frequency/seriousness of side effects, the drug’s impact on cognition, and effectiveness of systemic therapy; factors that can be due to intrinsic properties of the drug or possible interactions with other therapies [96]. For these reasons, the older AEDs (CBZ, PB and PHT) should no longer be considered first-line drugs in the treatment of seizures in patients with BTs; due to the fact that these AEDs can alter the metabolism of chemotherapeutic drugs resulting in their decreased efficacy. In this patient population, new generation drugs such as GBP, LCM, LTG, LEV, OXV, PGB, TPM, ZNS. are preferred because they have fewer drug interactions. Among the recently marketed drugs, LCM has demonstrated promising results and should be considered a possible treatment option. 

In conclusion, it is necessary to develop a customized treatment plan for each individual patient with BTRE, the goals of which should be complete seizure control, minimal or no side effects, and elimination of cognitive impairment and/or psychosocial problems. Despite significant improvements in efficacy and safety offered by the newer AEDs, they are not yet able to reverse the mechanisms of drug resistance. For this reason, the neurologist needs to keep in mind the enormous medical, social, and economic consequences not only of uncontrolled seizures, but also of choosing the wrong AED. 

#### Impact of Epilepsy and Related Therapies on Quality of Life 

The QoL for patients with BT is affected by many factors, the most significant being the various therapies undertaken (e.g. CT, radiation, surgery, support therapies, and AEDs), possible physical disability due to the tumor location, and possible neurocognitive disturbances. Also, the appearance of cognitive disturbances among patients with BTRE can be due to tumor location and CT as well as radiation. The QoL for patients with BTRE needs to be a primary objective, and together with the knowledge that epilepsy can significantly affect the long-term disability of the patient, the choice of AED must take into consideration the fact that while controlling seizures, the drug could have an effect on cognitive functioning, efficacy of systemic therapies, and the frequency of adverse events. Periodic neurological and neuropsychological check-ups are an important part of patient evaluation and of the patient-doctor feedback. They allow the monitoring of neurocognitive performances and possible collateral effects over time, and thus, enable the team of medical professionals to plan any necessary interventional strategies [20,96]. 

#### Prophylactic Antiepileptic Therapy

In 2000, the Guidelines of the American Academy of Neurology were published, with respect to prophylactic use of AEDs in patients newly diagnosed with BT [16]. These guidelines affirm that AEDs used prophylactically are unable to prevent the onset of seizures. For the lack of efficacy in preventing seizures and for the potential serious side effects that can be induced, prophylactic use of AEDs should not be considered a routine in this patient population. In the event that the treating physician should decide to treat BT patients who have not experienced seizures with AEDs in prophylaxis, the suggested practice is to suspend the AEDs within one week following surgery, especially in those patients who are stable or who have had adverse events related to the AED therapy [16,97]. 

#### Antiepileptic Therapy

Seeing as epilepsy affects the QoL of BT patients and creates a possibile long-term disability, either because of factors related to the epilepsy itself or to the drugs utilized for controlling seizures, the choice of AED therapy must take into consideration not only the drug’s efficacy for seizure control, but also possibile effects of the drug on important aspects of the patient’s daily life, for example: cognitive function, the efficacy of systemic therapies, and the frequency of SE.

For all of these reasons, the newer generation drugs, such as GBP, LCM, LTG, LEV, OXC, PGB, TPM, ZNS should be the preferred therapies, for this patient population, in that they have little or no enzyme induction, less drug-drug interactions, and fewer side effects. 

## CONCLUSION

The potential consequences of using the older generation of antiepileptic drugs (CBZ, PB, PHT) must be seen not only in the context of their potential to cause serious side effects but must also be seen in terms of their possible contribution to a reduction in the patient’s life expectancy, due to their negative impact on systemic therapies. In addition, systemic treatments can also interfere with the metabolism of these older drugs, thus reducing their plasma levels and consequently efficacy in BT patients. Instead, the newer AEDs, such as LTG, LEV, OXC, TPM and the older AED, VPA, can be considered first choice therapies in monotherapy, for all of the reasons previously discussed. These same AEDs can be considered as add-on, in addition to LCM, PGB, ZNS.

The therapeutic plan should take into consideration the following:

- Whether or not a specific anticancer therapy is needed.
- Whether or not a rapid titration is needed (e.g. in patients with a low life-expectancy, as is the case with brain metastases).

For some patients, secondary effects might be used for possible “positive” effects, for example: sedative effects (PGB or TPM) in patients who are agitated; mood elevation (OXC or LTG) in depressed patients. 

In addition, the team of healthcare professionale should create a relationship with BTRE patients that focuses on accompanying them and their family throughout the illness, offering not only medical support, but also the opportunity for patients to be heard and to be supported during medical and personal challenges and/or difficulties. 

Taking care of the patient with brain tumor-related epilepsy means listening to him/her, understanding his/her choices and respecting his/her priorities. Healthcare practitioners need to appreciate the fact that “taking care of” these patients does not mean “curing” as much as it means recognising and responding to the needs of each unique individual. 

## Figures and Tables

**Table 1.** (Modified by Yap KY *et al.* 2008) Interactions Between CT and AEDsth

CT			AEDs

Gemcitabine			No interactions expected
Mecloratamine			
Oxaliplatin			
Pemetrexed			
Rituximab			

Bleomicine			PHT
Capecitabine			
Carboplatin			
Citarabine			
Fluorouracile			

Anastrozole	Etoposide	Sorafenib	PHT
Cisplatin	Exemestane Gefitinib	Sunitinib	CBZ
Ciclofosfamide	Irinotecan Ixabepilone	Tamoxifen	PB
Dacarbazine	Letrozole	Temsirolimus	PRM
Docetaxel	Metotrexate	Toremifene	VPA
Doxorubicine	Paclitaxel	Vinca Alkaloid	
Epirubicine	Procarbazine	Vorinostat	
Erlotinib			

Vorinostat			TPM
			LEV metabolite
